# Respiratory Tract Infections and Antibiotic Resistance: A Protective Role for Vitamin D?

**DOI:** 10.3389/fnut.2021.652469

**Published:** 2021-03-25

**Authors:** Emma J. Derbyshire, Philip C. Calder

**Affiliations:** ^1^Nutritional Insight, London, United Kingdom; ^2^Faculty of Medicine, School of Human Development and Health, University of Southampton, Southampton, United Kingdom; ^3^National Institute for Health Research Southampton Biomedical Research Center, University Hospital Southampton National Health Service Foundation Trust and University of Southampton, Southampton, United Kingdom

**Keywords:** respiratory tract infections, antibiotic resistance, vitamin D, immunonutrition, prehabilitation

## Abstract

Upper and lower respiratory tract infections are among the most common infections globally, and in the United Kingdom, they account for about half of all oral antibiotics prescribed. Antibiotic overuse and the emergence of “superbugs” that are resistant to their effects is a global problem that is becoming a serious concern. Considering this, the potential role of immunonutrition as a “prehabilitation” in helping to tackle bacterial infections and reduce over-reliance on antibiotic usage is gaining interest. This narrative mini-review summarizes current knowledge on the roles of certain nutrients in helping to modulate immune function, with particular focus on vitamin D. Vitamin D supplementation appears to reduce the risk of acute respiratory tract infections and thus could have a valuable role to play in reducing over-reliance on antibiotics. Investment in high-quality trials is needed to further explore this field.

## Introduction

There has been an upsurge of novel bacterial, viral, and fungal respiratory pathogens that are becoming increasingly challenging to treat, with respiratory tract infections (RTIs) being exacerbated by antibiotic resistance of Gram-positive and Grain-negative bacteria (1). Acute respiratory tract infections (ARTIs), which include upper respiratory tract infections (URTIs), are, among adults, the most common cause of antibiotic prescription (2). In the United Kingdom, an examination of over eight million patient records from 587 general practices showed that URTIs accounted for around 31% of oral antibiotic prescriptions and lower respiratory tract infections (LRTIs) accounted for around 19% (3).

The very first antibiotic, salvarsan, was developed in 1910 while penicillin discovery by Alexander Fleming followed in 1928 (4). Multiple antibiotics have been discovered since then, but now, after about 100 years of the “antibiotic era,” fewer new antibiotics are being identified and significant antibiotic resistance has emerged (4). The World Health Organization considers that the unprecedented use of antibiotics and subsequent antimicrobial resistance (AMR) is currently one of the largest threats to global health, food security, and human development (5, 6).

In 2016, ARTIs were responsible for ~2.38 million deaths globally (7, 8). Within the European Union, 25,000 people have been estimated to die annually because of AMR with resultant societal costs of around 1.5 billion euros annually (9). By 2050, it has been estimated that some 10 million people globally could die annually as a result of AMR—with 390,000 Europeans estimated to be affected and even larger proportions of Asian (4,730,000) and African (4,140,000) populations (6). It has been further predicted that standard antibiotic treatments may no longer work, subsequently making infections more difficult to treat and control (10).

Given the high prevalence of ARTIs coupled with rising rates of AMR, novel approaches are needed for the future. The concept of “prehabilitation,” including the role of immunonutrition, could play a pivotal role in helping to both prevent and offset RTIs should these occur. Prehabilitation has been well defined elsewhere as: “*interventions that can help to improve patient's health in advance of being exposed to a physiological stressor so they are then better able to cope with that stress*” (11). This narrative mini-review describes how immunonutrition could become a valuable tool in conventional medicine. It focuses on ARTIs and vitamin D, for which there is an expanding body of evidence.

## Nutrition, Infection, and Immunity

The roles of nutrients in supporting the function of the immune system are numerous and varied, with an adequate and balanced supply of nutrients being required if a suitable immune response is to be mounted (12). The immune system protects the body against infectious agents and is composed of innate responses—the body's first lines of defense—and adaptive responses that generate immunological memory (13). It is known that a bidirectional relationship exists between nutrition, infection, and immunity with changes in one impacting on each of the others (14). Micronutrients (vitamins and minerals) have extended roles influencing and supporting every stage of the human immune response (13). Subsequently, deficiencies in one or more micronutrients can affect both innate and adaptive immunity, resulting in immunosuppression and exacerbating susceptibility to infections (13).

A host of nutrients have been implicated as being essential for immunocompetence, including vitamins A, B2, B6, B9 (folic acid), B12, C, D, E, and iron, zinc, selenium, copper, and magnesium (14). Vitamins A, C, D, E, and zinc are important for the structural and functional integrity of the body's external and mucosal barriers to invading pathogens (15). Cellular processes of both innate and adaptive immunity, such as cell differentiation and proliferation, phagocytosis, respiratory burst, killing activity, cytokine production, and antibody production are all dependent on suitable amounts of vitamins A, D, C, E, B6, and B12, folate, iron, zinc, copper, selenium, and magnesium (15).

This mini-review focuses on vitamin D, due to the growing body of evidence favoring a role for vitamin D in preventing ARTIs. Vitamin D augments host barrier epithelial integrity by reinforcing intercellular junctions (16). It has also been found to trigger antimicrobial peptide production, which exhibits direct pathogen-killing capacity (17). The vitamin D receptor is expressed on many immune cell types including B-cells, T-cells, and antigen-presenting cells (18–20). Furthermore, some immune cell types, including macrophages and dendritic cells, can synthesize the active form of vitamin D, 1,25-dihydroxyvitamin D3 (21). These two observations suggest a high importance for vitamin D within the immune system. Indeed, vitamin D deficiency results in impaired localized innate immunity and a defective antigen-specific cellular immune response, correlated with a higher susceptibility to infections (22). Vitamin D metabolites have also been found to influence the expression and secretion of pro-inflammatory chemokines and cytokines (23), and vitamin D promotes the production of anti-microbial peptides such as cathelicidin (21, 24).

An established body of evidence now shows that 1,25-dihydroxyvitamin D3 influences endothelial membrane stability and acts on multiple parts of the innate and adaptive immune responses (21). Low levels of 1,25-dihydroxyvitamin D3 correlate with an increased risk of developing several immune-related disorders including respiratory infection and COVID-19 (21). Vitamin D has further been found to be involved in pulmonary angiotensin-converting enzyme 2 expression and has the ability to reduce lung surface tension in COVID-19 (25). Other work suggests that vitamin D may induce progesterone-induced blocking factor and exert inhibitory effects on inflammation including the cytokine IL-6, which tend to be elevated in COVID-19 (26).

## Vitamin D and RTIs

A growing number of studies have investigated the role vitamin D on the occurrence of ARTIs. Table 1 summarizes evidence from meta-analyses and Table 2 summarizes evidence from RCTs, published in the last 5 years with a focus on adulthood, although some meta-analyses included extended age ranges.

**Table 1:** Summary of meta-analyses of vitamin D and RTIs with a focus on adults.

**References**	**Details of studies included**	**Study type**	**Infection definition/form**	**Publication focus**	**Main findings**
Jolliffe et al. (27)	45 RCTs (*n* = 73, 384)	Meta-analysis [Update: additional RCTs added to Martineau et al. (28) meta-analysis]	ARTIs—The definition of ARI encompassed URI, LRI, and ARI of unclassified location (i.e., infection of the upper and/or lower respiratory tract).	RCTs of vitamin D supplementation	Protective effects against ARTIs were seen in trials where vitamin D was given: ∘ Via a daily dosing regimen (OR 0.75, 95% CI 0.61–0.93) ∘ at daily dose equivalents of 400–1,000 IU (OR 0.70, 95% CI 0.55–0.89) ∘ for a duration of ≤ 12 months (OR 0.82, 95% CI 0.72–0.93)
			Form: Not Specified		
Pham et al. (29)	14 studies of ARTI risk (*n* = 78, 127)	Systematic review and meta-analysis of observational studies	ARTI, defined as an acute infection of the respiratory tract in either the lower or upper airway or with the location not specified. ARTI was either self-reported or clinically confirmed.	Vitamin D status	Serum 25(OH)D concentrations were inversely associated with risk and severity of ARTI (pooled OR 1.83, 95% CI 1.42–2.37 and OR 2.46, 95% CI 1.65–3.66 comparing the lowest with the highest 25(OH)D category, respectively)
	10 studies for trend analysis (*n* = 69, 048)				
	5 studies ARTI and vitamin D concentration (*n* = 37, 902)		Form: Not Specified		
Zhou et al. (30)	8 studies (*n* = 20, 966)	Meta-analysis of observational studies.	Pneumonia infection.	Vitamin D status	Community-acquired pneumonia patients with vitamin D deficiency [serum 25(OH)D levels <20 ng/ml] experienced a significantly increased risk of pneumonia (OR 1.64, 95% CI 1.00–2.67)
			Form: Not Specified		
Martineau et al. (28)	25 eligible RCTs (*n* = 11, 321; individuals aged 0 to 95 years)	Meta-analysis of RCTs	Classified as an upper respiratory tract infection, lower respiratory tract infection, and acute respiratory tract infection of unclassified location.	RCTs of vitamin D supplementation	Vitamin D supplementation lowered ARTI risk among all participants (OR 0.88 95% CI 0.81–0.96); effects were greater among those more deficient at baseline
			Form: Not Specified		
Gysin et al. (31)	15 RCTs (*n* = 7, 053)	Meta-analysis of RCTs	The first episode of clinical RTI was reported as cold/influenza-like illness and laboratory confirmed by standard microbiological methods.	RCTs of vitamin D3 supplementation	There was a 6% risk reduction of clinical RTIs with vitamin D3 supplementation, but this was not statistically significant (RR 0.94; 95% CI 0.88–1.00)
			Form: Bacterial		

*ARTIs, acute respiratory tract infection; CI, confidence interval; LRTI, lower respiratory tract infection; OR, odds ratio: RTI, respiratory tract infection; URTI, upper respiratory tract infection*.

**Table 2:** Summary of recent RCTs investigating the effect of vitamin D supplementation on RTIs in adults.

**References**	**Study population**	**Study type**	**Intervention**	**Infection definition/form**	**Main findings**
Arihiro et al. (32)	*n* = 223 patients with inflammatory bowel disease	6-month multicenter double-blind, placebo-controlled RCT	500 IU (12.5 μg) vitamin D or control daily	Influenza infection diagnosed using influenza virus test kits.	Incidence of URTI was significantly lower in the vitamin D group (RR 0.59; 95% CI, 0.35–0.98)
				Form: Viral	
Slow et al. (33)	*n* = 60 vitamin D group, *n* = 57 placebo	6-week randomized, double-blind, placebo-controlled trial	Single high-dose vitamin D3 (200,000 IU)	Pneumonia that has been acquired outside of a hospital or health care setting.	Vitamin D increased the complete resolution of pneumonia in participants with baseline vitamin D levels <25 nmol/L (OR 17.0, 95% CI 1.40–549.4), but this was of modest statistical significance (*p* = 0.043)
				Form: Not specified	
Jung et al. (34)	*n* = 25 male taekwondo athletes aged 19–22 years	4-week double-blind, placebo-controlled RCT	5,000 IU (125 μg) vitamin D3 or control daily	The Wisconsin Upper Respiratory Symptom Survey-11 (WURSS-11) was used.	Serum 25(OH)D levels increased by 256% and were inversely associated with total URTI symptoms (*r* = −0.435, *p* = 0.015)
				Form: Not specified	
Ramos-Martinez et al. (35)	*n* = 86 patients with asthma aged 18–50 years	6-month double-blind, placebo-controlled RCT	10 IU (0.25 μg) calcitriol (1,25-(OH)_2_D_3_) or control daily	Respiratory infections in asthmatic patients.	Vitamin D supplementation reduced RTIs and reduced airways colonization by pathogenic bacteria
				Form: Bacterial	
Shimizu et al. (36)	*n* = 428, aged 45–74 years	16-week double-blind, placebo-controlled RCT	400 IU (10 μg) vitamin D or control daily.	The Japanese version of Wisconsin Upper Respiratory Symptom Survey-21 (WURSS21) was used.	Vitamin D reduces the duration of URTI, the physical severity of URTI, and the quality of life when suffering from URTI
				Form: Not specified	
Ginde et al. (37)	*n* = 107 longer-term care residents, aged over 60 years	12-month double-blind, parallel group, randomized controlled phase II trial	High dose (3,000–4,000 IU/75–100 μg day) or standard dose (400–1,000 IU/10–25 μg day).	Measured both upper (common colds, sinusitis, pharyngitis, otitis media) and lower (acute bronchitis, influenza, pneumonia) ARIs that required medical attention	The high-dose group had 0.67 ARIs per person-year compared to 1.11 in the standard dose group (incidence rate ratio 0.60; 95% CI 0.38–0.94; *p*= 0.02)
				Form: Not specified	
He et al. (38)	*n* = 39 athletes during winter training	14-week placebo-controlled RCT	5,000 IU (125 μg) vitamin D or control daily	Measured changes in antimicrobial peptides.	Blood and salivary analyses showed that serum 25(OH)D levels increased by 130% and vitamin D increased SIgA and cathelicidin, which could improve resistance to respiratory infections
				Form: Bacterial	

*UPTI, upper respiratory tract infection; CI, confidence interval; OR odds ratio*.

Two meta-analyses focused on observational research (29, 30) and four focused on evidence from RCTs (27, 28, 31). Those collating observational findings found inverse relationships between serum 25-hydroxyvitamin D levels and risk and severity of ARTIs (29) and risk of community-acquired pneumonia (30). Meta-analyses pooling evidence from RCTs focused on findings from vitamin D supplementation programs represent a higher level of evidence since they can establish a cause-and-effect relationship. The largest meta-analysis included data from 45 RCTs (*n* = 73,384 subjects) concluding that daily dosing regimens providing 400–1,000 IU (10–25 μg) of vitamin D were most effective at protecting against ARTIs (27). Earlier meta-analyses reported similar findings: that vitamin D supplementation lowered ARTI risk (28), particularly among those with profound 25-hydroxyvitamin D deficiency at baseline (28). Focusing on vitamin D supplementation, a separate meta-analysis (15 RCTs, *n* = 7053) observed a 6% risk reduction of clinical RTIs, but this was not statistically significant and heterogeneity among the included studies was high (*I*^2^= 57%) (31).

Evidence from individual RCTs has reported similar findings. Five studies reported that vitamin D supplementation reduced the incidence (32, 35, 37), duration and severity (36), and symptoms (34) of RTIs. Among asthmatic patients, Ramos-Martinez et al. observed that vitamin D reduced RTIs, an effect that correlated with higher sputum levels of IL-10, IFN-γ, and cathelicidin LL-37 (35). Vitamin D dosages used among the different studies were highly variable, ranging from just 10 IU (0.25 μg daily) (35) up to 4,000 IU (100 μg daily) (37). Similarly, durations of RCTs were also wide-ranging, with the shortest being 4 weeks (34) and the longest, conducted in health care residents, being over a 12-month period (37).

Regarding pathological cause of infection, only four studies clearly specified whether these were bacterial (31, 35, 38) or viral (32). The remaining studies focused on the location, duration, and/or severity of RTIs, but their source was not clearly defined or diagnosed.

## Discussion and Conclusions

Presently, vitamin D guidelines in the United Kingdom have been set at 10 μg daily from October to March to keep bones, teeth, and muscles healthy (39). However, given updated meta-analytical evidence and a growing number of RCTs, combined with the global COVID-19 pandemic, it seems timely that this advice should be updated to encompass respiratory health with the required supplemental dose being re-evaluated. Clearly, vitamin D intakes should conform to recommended upper safety limits established by expert authorities with the European Food Safety Authority setting an upper limit of 100 μg/day for adults, including pregnant and lactating women (40). Equally, supplementation should always be in addition to the consumption of a healthy, varied, and well-balanced diet. Nevertheless, a more desirable level of intake of vitamin D taking the latest evidence into account would be 2,000 IU (50 μg) daily to reduce the risk of ARTIs (41).

Regarding antibiotic use, more clinical studies evaluating the impact of vitamin D are needed as an outcome alongside ARTI incidence, symptoms, and severity. One Cochrane review evaluated evidence from seven studies where vitamin D was used as an adjunct to antibiotics to treat pneumonia, but findings were inconclusive (42). In Sweden, vitamin D3 supplementation [1,500–1,600 IU (37.5–40 μg) daily over 12 months] was found to significantly reduce antibiotic usage—from 20 to 15 days per person (43). Equally, future studies should clearly define the origin of pathological RTIs. It is possible that different vitamin D dosing regimens may be warranted for viral and bacterial infections, but there is presently not enough evidence to draw firm conclusions on this.

AMR poses a threat to future global health, and the current COVID-19 pandemic is highly damaging to health, societies, and economies. Urgent responses are needed. Supporting the immune system of the population in advance of exposure to infections (i.e., “immune prehabilitation”) would reduce the number and severity of infections and reduce use of antibiotics. Vitamin D has multiple roles in supporting the immune system (21, 44, 45) and evidence from RCTs demonstrates that supplemental vitamin D reduces risk of acquiring RTIs (32, 35, 37) as well as their duration and severity (34, 36) and may reduce antibiotic use (43). There is also evidence that individuals with better vitamin D status are less likely to develop COVID-19 and severe COVID-19 (46–48). Given these observations, guidance of vitamin D intake should consider immune health, in addition to bone, tooth, and muscle health. Higher vitamin D intake in the population would reduce infections, result in infections being less severe and reduce use of antibiotics. The RCTs that form the current evidence base have highly variable designs including substantial differences in dose of vitamin D used, regularity of dosing, and duration of dosing. Thus, further clinical trials and meta-analytical approaches are warranted to clarify matters of dose, dosing regimen, and the precise relationship between vitamin D status and immune and respiratory health in different groups of the population including older people and different ethnicities.

## Author Contributions

ED and PC compiled, researched, wrote, and edited the review.

## Conflict of Interest

ED is the founder of Nutritional Insight. The remaining author declares that the research was conducted in the absence of any commercial or financial relationships that could be construed as a potential conflict of interest.

## References

[1] Zumla A Memish ZA Maeurer M Bates M Mwaba P Al-Tawfiq JA. Emerging novel and antimicrobial-resistant respiratory tract infections: new drug development and therapeutic options. Lancet Infect Dis. (2014) 14:1136–49. 10.1016/S1473-3099(14)70828-X 25189352 PMC7106460

[2] Harris A Hicks L Qaseem A. Appropriate antibiotic use for acute respiratory tract infection in adults: advice for high-value care from the American College of Physicians and the Centers for Disease Control and Prevention. High Value Care Task Force of the American College of Physicians and for the Centers for Disease Control and Prevention. Ann Intern Med. (2016) 164:425–34. 10.7326/M15-1840 26785402

[3] Palin V Molter A Belmonte M Ashcroft DM White A Welfare W. Antibiotic prescribing for common infections in UK general practice: variability and drivers. J Antimicrob Chemother. (2019) 74:2440–50. 10.1093/jac/dkz163 31038162 PMC6640319

[4] Hutchings MI Truman AW Wilkinson B. Antibiotics: past, present and future. Curr Opin Microbiol. (2019) 51:72–80. 10.1016/j.mib.2019.10.008 31733401

[5] WHO. Antibiotic Resistance. (2020). Available online at: https://www.who.int/news-room/fact-sheets/detail/antibiotic-resistance

[6] O'Neill J. Antimicrobial Resistance: Tackling a Crisis for the Health and Wealth of Nations. (2014). Available online at: https://amr-review.org/sites/default/files/AMR%20Review%20Paper%20-%20Tackling%20a%20crisis%20for%20the%20health%20and%20wealth%20of%20nations_1.pdf

[7] GBD 2016 Causes of Death Collaborators. Global, regional, and national age-sex specific mortality for 264 causes of death, 1980-2016: a systematic analysis for the Global Burden of Disease Study 2016 GBD 2016. Causes of Death Collaborators. Lancet. (2017) 390:1151–210. 10.1016/S0140-6736(17)32152-9 28919116 PMC5605883

[8] GBD 2016b Lower Respiratory Infections Collaborators. Estimates of the global, regional, national morbidity. mortality, and aetiologies of lower respiratory infections in 195 countries, 1990-2016: a systematic analysis for the Global Burden of Disease Study 2016. Lancet Infect Dis. (2018) 18:1191–210. 10.1016/S1473-3099(18)30310-4 30243584 PMC6202443

[9] HPRA. Health Products Regulatory Authority. Report on Antimicrobial Resistance, Dublin (2016).

[10] WHO. Antimicrobial Resistance. Global Report on Surveillance, Switzerland (2014).

[11] Silver J. Prehabilitation could save lives in a pandemic. BMJ. (2020) 369:m1386. 10.1136/bmj.m1386 32253187

[12] Calder P. Nutrition, immunity and COVID-19. BMJ Nutr Prev Health. (2020) 3:74–92. 10.1136/bmjnph-2020-000085 PMC7295866 33230497

[13] Pecora F Persico F Argentiero A Neglia C Esposito S. The role of micronutrients in support of the immune response against viral infections. Nutrients. (2020) 12:3198. 10.3390/nu12103198 33092041 PMC7589163

[14] Maggini S Pierre A Calder PC. Immune function and micronutrient requirements change over the life course. Nutrients. (2018) 10:1531. 10.3390/nu10101531 30336639 PMC6212925

[15] Gombart AF Pierre A Maggini S. A review of micronutrients and the immune system-working in harmony to reduce the risk of infection. Nutrients. (2020) 12:236. 10.3390/nu12010236 31963293 PMC7019735

[16] Fakhoury HMA Kvietys PR AlKattan W Anouti FA Elahi MA Karras SN. Vitamin D and intestinal homeostasis: barrier, microbiota, immune modulation. J Steroid Biochem Mol Biol. (2020) 200:105663. 10.1016/j.jsbmb.2020.105663 32194242

[17] Korf H Decallonne B Mathieu C. Vitamin D for infections. Curr Opin Endocrinol Diabetes Obes. (2014) 21:431–6. 10.1097/MED.0000000000000108 25354043

[18] Aranow C. Vitamin D and the immune system. J Investig Med. (2011) 59:881–6. 10.2310/JIM.0b013e31821b8755 PMC3166406 21527855

[19] Borges MC Martini LA Rogero MM. Current perspectives on vitamin D, immune system, chronic diseases. Nutrition. (2011) 27:399–404. 10.1016/j.nut.2010.07.022 20971616

[20] Mora JR Iwata M Andrian von UH. Vitamin effects on the immune system: vitamins A and D take centre stage. Nat Rev Immunol. (2008) 8:685–98. 10.1038/nri2378 19172691 PMC2906676

[21] Charoenngam N Holick MF. Immunologic effects of vitamin D on human health and disease. Nutrients. (2020) 12:2097. 10.3390/nu12072097 PMC7400911 32679784

[22] Wintergerst ES Maggini S Hornig DH. Contribution of selected vitamins and trace elements to immune function. Ann Nutr Metab. (2007) 51:301–23. 10.1159/000107673 17726308

[23] Greiller CL Martineau AR. Modulation of the immune response to respiratory viruses by vitamin D. Nutrients. (2015) 7:4240–70. 10.3390/nu7064240 26035247 PMC4488782

[24] Chung C Silwal P Kim I Modlin RL Jo EK. Vitamin D-Cathelicidin Axis: at the Crossroads between Protective Immunity and Pathological Inflammation during Infection. Immune Netw. (2020) 20:e12. 10.4110/in.2020.20.e12 32395364 PMC7192829

[25] Ebadi M Montano-Loza AJ. Perspective: improving vitamin D status in the management of COVID-19. Eur J Clin Nutr. (2020) 74:856–9. 10.1038/s41430-020-0661-0 32398871 PMC7216123

[26] Orru B Szekeres-Bartho J Bizzarri M Spiga AM Unfer V. Inhibitory effects of Vitamin D on inflammation and IL-6 release. A further support for COVID-19 management? Eur Rev Med Pharmacol Sci. (2020) 24:8187–93. 10.26355/eurrev_202008_22507 32767348

[27] Jolliffe DA Camargo CA Sluyter JD Aglipay M Aloia JF Ganmaa D. Vitamin D supplementation to prevent acute respiratory infections: systematic review and meta-analysis of aggregate data from randomised controlled trials. medRxiv [Preprint]. (2020). 10.1101/2020.07.14.20152728 33798465

[28] Martineau AR Jolliffe DA Greenberg L Aloia JF Bergman P Dubnov-Raz G. Vitamin D supplementation to prevent acute respiratory infections: individual participant data meta-analysis. Health Technol Assess. (2019) 23:1–44. 10.3310/hta23020 30675873 PMC6369419

[29] Pham H Rahman A Majidi A Waterhouse M Neale RE. Acute respiratory tract infection and 25-hydroxyvitamin d concentration: a systematic review and meta-analysis. Int J Environ Res Public Health. (2019) 16:3020. 10.3390/ijerph16173020 31438516 PMC6747229

[30] Zhou YF Luo BA Qin LL. The association between vitamin D deficiency and community-acquired pneumonia: a meta-analysis of observational studies. Medicine (Baltimore). (2019) 98:e17252. 10.1097/MD.0000000000017252 31567995 PMC6756683

[31] Gysin DV Dao D Gysin CM Lytvyn L Loeb M. Effect of Vitamin D3 Supplementation on respiratory tract infections in healthy individuals: a systematic review and meta-analysis of randomized controlled trials. PLoS One. (2016) 11:e0162996. 10.1371/journal.pone.0162996 27631625 PMC5025082

[32] Arihiro S Nakashima A Matsuoka M Suto S Uchiyama K Kato T. Randomized trial of vitamin D supplementation to prevent seasonal influenza and upper respiratory infection in patients with inflammatory bowel disease. Inflamm Bowel Dis. (2019) 25:1088–95. 10.1093/ibd/izy346 30601999 PMC6499936

[33] Slow S Epton M Storer M Thiessen R Lim S Wong J. Effect of adjunctive single high-dose vitamin D3 on outcome of community-acquired pneumonia in hospitalised adults: the VIDCAPS randomised controlled trial. Sci Rep. (2018) 8:13829. 10.1038/s41598-018-32162-2 30218062 PMC6138743

[34] Jung HC Seo MW Lee S Kim SW Song JK. Vitamin D(3) Supplementation reduces the symptoms of upper respiratory tract infection during winter training in vitamin D-insufficient taekwondo athletes: a randomized controlled trial. Int J Environ Res Public Health. (2018) 15:2003. 10.3390/ijerph15092003 30223447 PMC6164435

[35] Ramos-Martinez E Lopez-Vancell MR Fernandez de Cordova-Aguirre JC Rojas-Serrano J Chavarria A Velasco-Medina A. Reduction of respiratory infections in asthma patients supplemented with vitamin D is related to increased serum IL-10 and IFNgamma levels and cathelicidin expression. Cytokine. (2018) 108:239–46. 10.1016/j.cyto.2018.01.001 29402723

[36] Shimizu Y Ito Y Yui K Egawa K Orimo H. Intake of 25-hydroxyvitamin D3 reduces duration and severity of upper respiratory tract infection: a randomized, double-blind, placebo-controlled, parallel group comparison study. J Nutr Health Aging. (2018) 22:491–500. 10.1007/s12603-017-0952-x 29582888 PMC5866826

[37] Ginde AA Blatchford P Breese K Zarrabi L Linnebur SA Wallace JI. High-Dose Monthly vitamin D for prevention of acute respiratory infection in older long-term care residents: a randomized clinical trial. J Am Geriatr Soc. (2017) 65:496–503. 10.1111/jgs.14679 27861708 PMC5357189

[38] He CS Fraser WD Tang J Brown K Renwick S Rudland-Thomas J. The effect of 14 weeks of vitamin D3 supplementation on antimicrobial peptides and proteins in athletes. J Sports Sci. (2016) 34:67–74. 10.1080/02640414.2015.1033642 25861808

[39] NICE NICE PHE and SACN Publish Rapid COVID-19 Guidance on Vitamin D (2020). Available online at: https://www.nice.org.uk/news/article/nice-phe-and-sacn-publish-rapid-covid-19-guidance-on-vitamin-d

[40] EFSA European Food Safety Authority. Scientific opinion on the tolerable upper intake level of vitamin D. EFSA Panel on Dietetic Products, Nutrition and Allergies. EFSA J. (2012) 10:2813. 10.2903/j.efsa.2012.2813

[41] Calder PC Carr AC Gombart AF Eggersdorfer M. Optimal nutritional status for a well-functioning immune system is an important factor to protect against viral infections. Nutrients. (2020) 12:1181. 10.3390/nu12041181 32756516 PMC7469053

[42] Das RR Singh M Naik SS. Vitamin D as an adjunct to antibiotics for the treatment of acute childhood pneumonia. Cochrane Database Syst Rev. (2018) 7:CD011597. 10.1002/14651858.CD011597.pub2 30024634 PMC6513535

[43] Norlin AC Hansen S Wahren-Borgstrom E Granert C Bjorkhem-Bergman L Bergman P. Vitamin D3 supplementation and antibiotic consumption - results from a prospective, observational study at an immune-deficiency unit in Sweden. PLoS One. (2016) 11:e0163451. 10.1371/journal.pone.0163451 27657724 PMC5033385

[44] Siddiqui M Manansala JS Abdulrahman HA Nasrallah GK Smatti MK Younes N. Immune modulatory effects of vitamin D on viral infections. Nutrients. (2020) 12:2879. 10.3390/nu12092879 32967126 PMC7551809

[45] Hewison M Vitamin D and immune function: an overview. Proc Nutr Soc. (2012) 71:50–61. 10.1017/S0029665111001650 21849106

[46] Grant WB Lahore H McDonnell SL Baggerly CA French CB Aliano JL. Evidence that vitamin D supplementation could reduce risk of influenza and COVID-19 infections and deaths. Nutrients. (2020) 12:988. 10.3390/nu12040988 32252338 PMC7231123

[47] Laird E Rhodes J Kenny RA. Vitamin D and inflammation: potential implications for severity of Covid-19. Ir Med J. (2020) 113:81. 32603576

[48] Kara M Ekiz T Ricci V Kara O Chang KV Ozcakar L. ‘Scientific Strabismus' or two related pandemics: coronavirus disease and vitamin D deficiency. Br J Nutr. (2020) 124:736–41. 10.1017/S0007114520001749 32393401 PMC7300194

